# Medical image pretraining-based transfer learning for generalizable and robust diagnosis of bone tumors on radiographs: a multi-center study

**DOI:** 10.1186/s13244-026-02271-y

**Published:** 2026-04-07

**Authors:** Zhihui Li, Hanqi Wang, Gang Wei, Xiaochuan Geng, Yong Lu, Wei Xia, Caifang Ni

**Affiliations:** 1 Department of Interventional Radiology, The First Affiliated Hospital of Soochow University, Suzhou, China; 2 Department of Radiology, Ruijin Hospital, Shanghai Jiao Tong University School of Medicine, Shanghai, China; 3 Clinical Neuroscience Center, Affiliated Ruijin Hospital, Shanghai Jiao Tong University School of Medicine, Shanghai, China; 4 Department of Radiology, Renji Hospital, Shanghai Jiao Tong University School of Medicine, Shanghai, China; 5 Faculty of Medical Imaging Technology, College of Health Science and Technology, Shanghai Jiao Tong University School of Medicine, Shanghai, China

**Keywords:** Bone tumor, Radiograph, Transfer learning, Artificial intelligence

## Abstract

**Objectives:**

To develop a generalizable and robust deep learning model for bone tumor classification in radiographs by leveraging domain-specific medical image pretraining.

**Materials and methods:**

This retrospective multi-center study included 2338 patients with histopathologically confirmed bone tumors from four centers. Four hundred seventy-one patients from one center were used for model development, and 1867 patients from the other three centers were used for the external test. Deep learning models (ResNet50 and InceptionV3) were developed using transfer learning with weights from either RadImageNet (medical images) or ImageNet (natural images). A radiomics model based on ElasticNet was also built. Model performance was evaluated using the area under the curve (AUC), and the paired DeLong test was used to evaluate statistical significance between AUCs. Robustness was assessed through tumor bounding box perturbation experiments. Gradient-weighted class activation mapping (Grad-CAM) was performed to localize the key area highlighted by the model for enhancing interpretability.

**Results:**

ResNet50 pretrained on RadImageNet demonstrated improved performance on external test sets (AUC = 0.738, 95% CI: 0.714–0.762), outperforming ImageNet-pretrained models (ResNet50: AUC = 0.669, 95% CI: 0.639–0.699, *p* < 0.001; InceptionV3: AUC = 0.677, 95% CI: 0.647–0.708, *p* < 0.001) and the radiomics model (AUC = 0.518, 95% CI: 0.487–0.548, *p* < 0.001). RadImageNet-pretrained models showed higher stability under tumor bounding box perturbation conditions (*p* < 0.001), and appropriately focused on diagnostically relevant regions in correctly classified cases.

**Conclusion:**

The deep learning model pretrained on domain-specific medical images demonstrated improved performance and robustness compared to the radiomics and natural image-pretrained models for bone tumor classification on radiographs.

**Critical relevance statement:**

Domain-specific medical image pretraining enhanced deep learning model performance and robustness over radiomics and natural image approaches in bone tumor classification on radiographs.

**Key Points:**

- Domain-specific medical image pretraining (RadImageNet) significantly outperforms natural image pretraining (ImageNet) for bone tumor classification on radiographs.
- Deep learning models pretrained on medical images demonstrate superior performance compared to radiomics approaches for bone tumor classification.
- AI assistance effectiveness varies among radiologists, with performance improvements depending on individual experience and receptiveness to AI support.

**Graphical Abstract:**

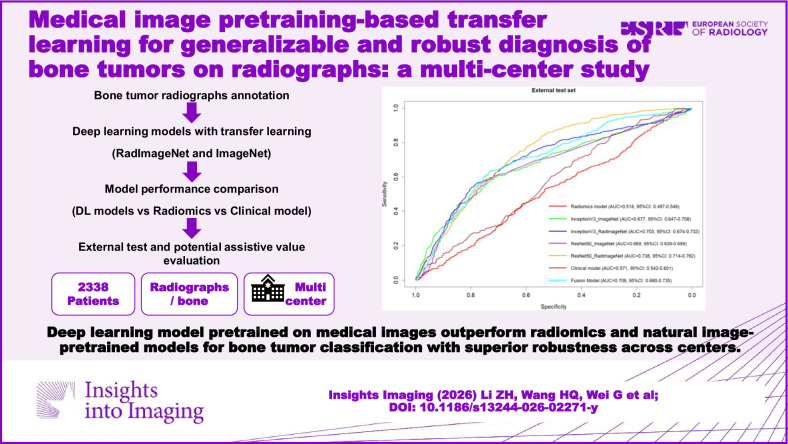

## Introduction

Bone tumors represent a diverse group of neoplasms with distinct biological behaviors, ranging from benign lesions with a favorable prognosis to highly aggressive malignancies such as osteosarcoma and chondrosarcoma [1, 2]. Accurate differentiation between benign and non-benign subtypes is crucial for clinical decision-making. Benign tumors often require only conservative management or minor surgical intervention, whereas other variants necessitate therapies including chemotherapy, radiotherapy, and extensive surgery [3]. Misclassification may lead to unnecessary biopsies, increased healthcare costs, delayed treatment, or even life-threatening consequences [4].

Radiographic imaging remains the first-line diagnostic modality for bone tumors due to its accessibility, cost-effectiveness, and ability to depict key morphological features such as bone destruction patterns, periosteal reactions, and soft-tissue involvement [5]. However, the low incidence of bone tumors limits widespread expertise among radiologists, resulting in substantial inter-observer variability and potential misdiagnosis [6]. Moreover, manual interpretation is time-consuming and subject to human bias, especially when distinguishing overlapping radiographic features across tumor types [7]. Furthermore, bone tumors are frequently encountered as incidental findings by general radiologists in routine clinical practice. In these community hospital and general practice settings, where immediate access to specialized consultation may not be available, there is a critical need for reliable decision support tools to assist in the initial triage and management decisions. This represents a high-value clinical application where AI-assisted diagnosis could significantly improve patient care and workflow efficiency.

Recent advances in artificial intelligence (AI), particularly deep learning (DL), have shown promise in improving diagnostic accuracy and efficiency in musculoskeletal imaging [8]. DL-based models can automatically extract image features without requiring labor-intensive manual segmentation of the tumor boundary, offering advantages over traditional radiomics approaches that rely on handcrafted features [9]. However, most existing studies apply natural-image pretraining (e.g., ImageNet) to initialize convolutional neural networks, which introduces a domain gap due to the significant differences between medical and natural images [10]. Furthermore, many AI models for bone tumor classification are validated with small datasets, leading to concerns about overfitting and limited generalizability [11].

To address these limitations, recent efforts have focused on domain-specific pretraining using large-scale medical image repositories. RadImageNet, a publicly available dataset containing 1.35 million annotated medical images from 131,872 patients, has been proposed to bridge the domain gap by providing pretrained model weights optimized for medical imaging tasks [12]. In addition, large-scale multi-center validation has become increasingly important to ensure robustness and clinical applicability [13]. Clinically critical concerns such as model interpretability—highlighting diagnostically relevant regions—and stability testing under realistic variations in tumor localization remain largely unaddressed, despite being essential considerations for real-world clinical implementation [14].

Therefore, this multi-center study developed and validated DL models using RadImageNet pretraining for bone tumor classification on radiographs. We compared performance against conventional radiomics and ImageNet-pretrained models across four centers. Model robustness, interpretability, and potential assistive value were also evaluated.

## Materials and methods

This retrospective multi-center study was conducted in accordance with the Declaration of Helsinki and was approved by the Ethics Committee of Ruijin Hospital, Shanghai Jiao Tong University School of Medicine (No. 2024-157), and the requirement for informed consent was waived due to the retrospective nature of the study.

### Patient population and data collection

**Fig. 1:**
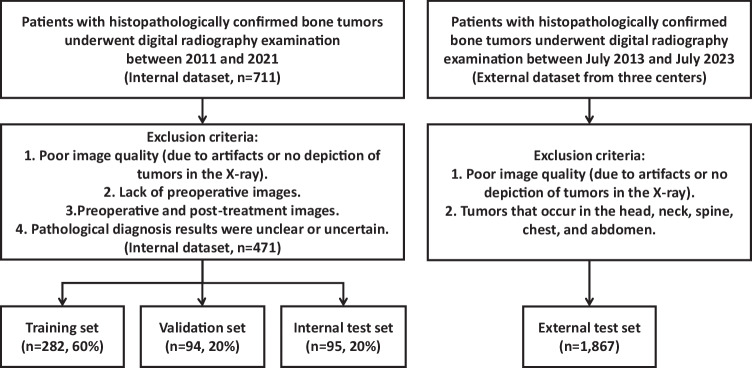
The flow diagram of patient enrollment

We included 2338 patients with histopathologically confirmed bone tumors from four centers. The internal dataset comprised 471 patients from our hospital between 2011 and 2021, while the external dataset included 1867 patients with tumors from three additional centers between July 2013 and July 2023 participating in the bone tumor X-ray radiograph dataset (BTXRD) [15]. Inclusion and exclusion criteria were shown in Fig. 1. Patients selected for this study had received a diagnosis of either benign tumors or non-benign tumors (including malignant and intermediate bone tumors). All tumors were pathologically confirmed and grouped using the 5th World Health Organization classification of soft tissue and bone tumors published in 2020 [16].

The internal dataset was randomly divided into training (*n* = 282, 60%), validation (*n* = 94, 20%), and internal test sets (*n* = 95, 20%) using stratified sampling to maintain the proportion of benign and non-benign cases. The BTXRD dataset served as an independent external test set. Since our center is not a contributor to the BTXRD repository, and the datasets originate from geographically distinct centers, the training/validation/internal test sets and the external test set are fully independent.

### Image acquisition and annotation

For the internal dataset, radiographs were acquired using a SIEMENS digital radiography system with imaging parameters including tube voltage 50–120 kVp, tube current-time product 2–10 mAs, and source-to-image distance 100–120 cm. Both anteroposterior and lateral views were obtained when clinically indicated. To ensure dataset consistency and avoid redundancy, only one representative radiographic image (the view best demonstrating the lesion) was selected for each patient in the internal cohort. Therefore, the dataset maintains a strict one-to-one correspondence between images and patients, inherently preventing data leakage associated with multi-view dependencies. All radiographs were independently reviewed by two musculoskeletal radiologists with more than 10 years of experience. Tumor regions were manually annotated with ITK-SNAP software [17] (version 4.0, https://www.itksnap.org/) encompassing the entire lesion, including areas of bone destruction, periosteal reaction, and associated soft tissue masses. Discrepancies were resolved through consensus review with a third senior radiologist. The external dataset BTXRD provides the annotated tumor region and bounding boxes.

### Model development

**Fig. 2:**
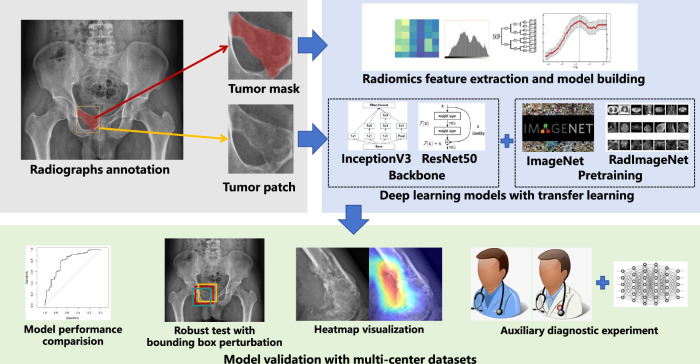
The overall workflow of this study. The tumor region with mask was used for radiomics modelling, and the tumor patch was used for DL model development. The DL pipeline utilizes InceptionV3 and ResNet50 architectures with transfer learning from both natural image (ImageNet) and medical image (RadImageNet) domains. Model validation includes performance comparison, robustness evaluation using bounding box perturbation, explainability through heatmap visualization, and potential assistive value assessment through auxiliary diagnostic experiments, all conducted across multi-center datasets

The overall workflow of this study was illustrated in Fig. 2. We implemented two DL model architectures with proven effectiveness in medical image analysis. ResNet50 addresses the vanishing gradient problem through skip connections, enabling training of deeper networks with 50 layers containing residual blocks with bottleneck designs [18]. InceptionV3 incorporates inception modules with multiple convolutional kernel sizes processed in parallel, enabling efficient multi-scale feature extraction through inception modules [19].

In this study, we employed two distinct pretraining approaches to investigate domain transfer effectiveness. Models were initialized with weights pretrained either on the ImageNet dataset containing 14 million natural images across 1000 categories [20], representing the conventional transfer learning approach [10], or on RadImageNet, a medical imaging-specific dataset comprising 1.35 million annotated medical images with 165 labels from 131,872 patients, specifically curated to address the domain gap between natural and medical images [12].

The pretrained models were then fine-tuned to classify the radiographs of bone tumors. Tumor bounding boxes were generated from the annotated tumor regions, and tumor patches were extracted from the bounding boxes. The tumor patches then underwent preprocessing, including resizing to 224 × 224 pixels (ResNet50) or 299 × 299 pixels (InceptionV3) and intensity normalization of [0, 1]. Data augmentation techniques were applied during training to enhance model generalization. Tumor patches were subjected to random horizontal and vertical flipping to simulate different tumor orientations. Affine transformations were applied with rotation up to 10 degrees, translation up to 20% in both horizontal and vertical directions, and random scaling between 80% and 120% of the original size to mimic natural variations in tumor imaging presentations.

Models were fine-tuned with a batch size of 128 using the Adam optimizer with an initial learning rate of 4 × 10^−3^. We employed Focal Loss to address class imbalance by focusing on hard-to-classify examples [21]. A ReduceLROnPlateau scheduler monitored the model performance in the validation set, reducing the learning rate by a factor of 0.618 after 10 epochs without improvement. L2 regularization was implemented by adding the scaled L2 norm of model parameters to the loss function, helping prevent overfitting while preserving model discriminative capacity. All experiments were conducted with NVIDIA Tesla A100 GPU and PyTorch framework (version 2.4.1).

For comparison, a radiomics model was constructed using 455 features extracted from the tumor region with a mask via PyRadiomics (version 3.1.0) [22]. These features included 18 first-order statistics, 14 shape-based parameters, 73 texture features derived from multiple matrices, and 364 wavelet-transformed features. The remaining features then underwent selection via ElasticNet [23] using grid search to identify optimal alpha values (ranging from 0.1 to 0.9) for radiomics model development through 10-fold cross-validation.

A clinical model incorporating patient age, gender, and tumor location was built using ElasticNet. A fusion model was further built by combining the best model predictions with clinical features through ElasticNet.

### Model evaluation

Model performance was assessed using the area under the receiver operating characteristic curve (AUC). Statistical significance between AUCs was assessed with the paired DeLong test. To control for Type I error inflation, the Bonferroni correction was applied specifically to the pairwise comparisons between pretraining strategies (RadImageNet vs ImageNet). Accuracy, sensitivity, and specificity were calculated based on the prediction threshold corresponding to the maximum Youden Index in the training set.

Model robustness under simulated real-world localization variability was tested by perturbation experiments of randomly displacing tumor bounding box coordinates (± 30% in *x*/*y* directions) 50 times per case, analyzing AUC distribution with Wilcoxon signed-rank tests.

Gradient-weighted class activation mapping (Grad-CAM) generated attention heatmaps visualizing regions contributing to predictions [24].

An auxiliary diagnostic experiment involved two attending musculoskeletal radiologists independently reviewing an internal test set and 100 randomly selected cases from an external test set, first without and then with model assistance (predictions and heatmaps), and the washout period was two weeks. Radiologist 1 had 10 years of experience in musculoskeletal imaging, and Radiologist 2 had 15 years of experience. Diagnostic performance changes in AUC were quantified using the paired DeLong test. The precision and clinical relevance of the findings were further assessed by examining the 95% confidence intervals of the AUC differences in relation to a clinically meaningful performance improvement threshold (defined as ΔAUC > 0.05).

### Statistical analysis

Continuous variables were expressed as mean ± standard deviation or median (interquartile range) based on normality testing (Shapiro–Wilk), with group comparisons using Student’s *t*-test or Mann–Whitney *U*-test. Categorical variables were compared using chi-square or Fisher’s exact test. A two-sided *p* value < 0.05 was considered statistically significant. Statistical analyses were performed using R software (version 4.4.0). The code of this study is available on GitHub (https://github.com/xiawei999000/bone_tumor).

## Results

### Patient characteristics

The study included 2338 patients from two independent datasets: 471 (20.1%) from the internal dataset and 1867 (79.9%) from the external dataset. Table 1 presents the clinical characteristics stratified by dataset. While gender distribution was comparable between cohorts (Male: 54.4% vs 57.6%, χ^2^ = 1.47, *p* = 0.226), patients in the internal dataset were significantly older than those in the external dataset (37.5 ± 21.0 vs 31.5 ± 21.2 years, *t* = 5.53, *p* < 0.001).

**Table 1:** The comparison of clinical characteristics of patients between the internal and external datasets

Characteristics	Internal dataset	External dataset	*p*
Age (years)	37.5 ± 21	31.5 ± 21.2	< 0.001
Gender (male)	256 (54.4%)	1075 (57.6%)	0.226
Tumor type (benign)	173 (36.7%)	1432 (76.7%)	< 0.001
Osteochondroma	28 (5.9%)	754 (40.4%)	< 0.001
Simple bone cyst	8 (1.7%)	206 (11.0%)	< 0.001
Other benign tumor	137 (29.1%)	472 (25.3%)	0.093
Tumor type (non-benign)	298 (63.3%)	435 (23.3%)	< 0.001
Giant cell tumor	32 (6.8%)	93 (5.0%)	0.118
Osteosarcoma	108 (22.9%)	298 (16.0%)	< 0.001
Other non-benign tumors	158 (33.5%)	44 (2.4%)	< 0.001
Upper limb	113 (24.0%)	449 (24.1%)	0.979
Hand	20 (4.2%)	93 (5.0%)	0.506
Ulna	4 (0.9%)	39 (2.1%)	0.074
Radius	11 (2.3%)	69 (3.7%)	0.147
Humerus	78 (16.6%)	248 (13.3%)	0.066
Lower limb	311 (66.0%)	1347 (72.2%)	0.009
Foot	16 (3.4%)	90 (4.8%)	0.184
Tibia	71 (15.1%)	518 (27.8%)	< 0.001
Fibula	22 (4.7%)	123 (6.6%)	0.123
Femur	202 (42.9%)	616 (33.0%)	< 0.001
Axial skeleton	47 (10.0%)	71 (3.8%)	< 0.001
Pelvis	36 (7.6%)	71 (3.8%)	< 0.001
Spine	11 (2.4%)	0 (0%)	< 0.001

Pathological distributions differed substantially between the two datasets. The external dataset contained a significantly higher proportion of benign tumors (76.7% vs 36.7%, χ^2^ = 281.45, *p* < 0.001), primarily driven by a higher prevalence of osteochondroma (40.4% vs 5.9%, χ^2^ = 210.87, *p* < 0.001) and simple bone cysts (11.0% vs 1.7%, χ^2^ = 41.13, *p* < 0.001). Conversely, the internal dataset was enriched with non-benign cases (63.3% vs 23.3%, *p* < 0.001), with a higher proportion of osteosarcoma (22.9% vs 16.0%, χ^2^ = 12.15, *p* < 0.001) and other non-benign tumors (33.5% vs 2.4%, χ^2^ = 459.9, *p* < 0.001). Notably, no significant differences were observed in the proportions of other benign tumors (χ^2^ = 2.82, *p* = 0.093) and giant cell tumors (χ^2^ = 2.44, *p* = 0.118), indicating partial overlap in pathological spectra.

Tumor location patterns also varied markedly. Overall upper limb involvement was similar (24.0% vs 24.1%, χ^2^ = 0.001, *p* = 0.979). In contrast, lower limb tumors were more frequent in the external dataset (66.0% vs 72.2%, χ^2^ = 6.83, *p* = 0.009), particularly affecting the tibia (15.1% vs 27.8%, χ^2^ = 30.0, *p* < 0.001), whereas femoral lesions were more common in the internal dataset (42.9% vs 33.0%, χ^2^ = 16.2, *p* < 0.001). Axial skeleton involvement was significantly higher in the internal dataset (10.0% vs 3.8%, χ^2^ = 29.93, *p* < 0.001), with spinal lesions present exclusively in the internal cohort (2.4% vs 0%). These substantial differences in demographics, tumor subtypes, and anatomical distributions between training and testing datasets provide a rigorous evaluation framework for assessing the model’s generalizability across diverse clinical populations.

To quantitatively evaluate the domain shift, the images from internal and external datasets were normalized by subtracting the minimum and dividing by the range, then four key metrics were calculated to reflect histogram distributions: (1) mean intensity: reflecting global brightness/exposure differences. (2) contrast (pixel standard deviation): reflecting dynamic range differences. (3) entropy: reflecting texture complexity and noise levels. (4) aspect ratio: reflecting differences in field-of-view and cropping strategies. The independent sample T test (Student’s *t*-test or Welch’s *t*-test according to the homogeneity of variance) is used to compare the differences between the two data sets, and calculate Cohen’s d effect to evaluate the difference. And the results were detailed in Table S1 and the histogram distributions were illustrated in Fig. S1. While mean intensity (brightness) remained consistent (*p* = 0.468, Cohen’s *d* = 0.04), reflecting standardized exposure levels, a substantial divergence was observed in image contrast. The external dataset exhibited significantly higher contrast compared to the internal set (0.215 vs 0.151, *p* < 0.001), with a very large effect size (Cohen’s *d* = 1.30).

### Model performance

Table 2 present the complete radiomics model performance metrics. The radiomics model employed 11 selected features from the original 455 extracted features, predominantly consisting of wavelet transformation features (8/11), and the detailed feature names and corresponding coefficients were provided in Table S2. Furthermore, radiomics feature importance analysis based on SHAP (SHapley Additive exPlanations) values was added, and the feature importance ranking plots were illustrated in Fig. S3.

**Table 2:** The performance of models on internal and external tests

Models	Internal test				External test			
	AUC	ACC	SEN	SPE	AUC	ACC	SEN	SPE
Radiomics model	0.694 (0.587–0.800)	0.653 (0.553–0.741)	0.550 (0.425–0.669)	0.829 (0.673–0.919)	0.518 (0.487–0.548)	0.529 (0.507–0.552)	0.533 (0.486–0.580)	0.528 (0.502–0.554)
InceptionV3_ImageNet	0.724 (0.622–0.826)	0.653 (0.584–0.747)	0.583 (0.449–0.709)	0.771 (0.599–0.596)	0.677 (0.647–0.708)	0.694 (0.672–0.714)	0.582 (0.534–0.628)	0.728 (0.704–0.715)
InceptionV3_RadImageNet	0.774 (0.678–0.870)	0.737 (0.636–0.822)	0.833 (0.715–0.917)	0.571 (0.394–0.737)	0.703 (0.674–0.732)	0.655 (0.633–0.677)	0.692 (0.646–0.735)	0.644 (0.618–0.669)
ResNet50_ImageNet	0.723 (0.612–0.834)	0.716 (0.614–0.804)	0.800 (0.677–0.892)	0.571 (0.394–0.737)	0.669 (0.639–0.699)	0.686 (0.664–0.707)	0.607 (0.559–0.653)	0.709 (0.685–0.733)
ResNet50_RadImageNet	0.785 (0.686–0.884)	0.768 (0.671–0.849)	0.800 (0.677–0.892)	0.714 (0.537–0.854)	0.738 (0.714–0.762)	0.707 (0.686–0.728)	0.572 (0.524–0.619)	0.748 (0.725–0.770)
Clinical model	0.677 (0.568–0.787)	0.568 (0.468–0.663)	0.317 (0.213–0.442)	1.000 (0.901–1.000)	0.571 (0.542–0.601)	0.418 (0.396–0.441)	0.848 (0.812–0.879)	0.288 (0.265–0.312)
Fusion model	0.785 (0.685–0.886)	0.779 (0.686–0.851)	0.833 (0.720–0.907)	0.686 (0.520–0.814)	0.708 (0.680–0.735)	0.705 (0.684–0.726)	0.637 (0.591–0.681)	0.726 (0.703–0.749)

ACC, SEN, and SPE were calculated based on the prediction threshold corresponding to the maximum Youden Index in the training set

*AUC* area under the receiver operating characteristic curve, *ACC* accuracy, *SEN* sensitivity, *SPE* specificity

Radiomics model achieved moderate performance on the internal test set (AUC = 0.694, 95% CI: 0.587–0.800) but showed substantially reduced performance on the external test set (AUC = 0.518, 0.487–0.548), indicating poor generalizability.

All DL models outperformed the radiomics model, particularly on the external test set, where generalizability is essential. The ResNet50 architecture pretrained on RadImageNet (ResNet50_RadImageNet) demonstrated the highest performance with AUC = 0.785 (95% CI: 0.686–0.884) on the internal test set and AUC = 0.738 (95% CI: 0.714–0.762) on the external test set. The model inference time was 0.050 ± 0.038 s per case on the GPU. Comparing pretraining approaches, models pretrained on RadImageNet consistently outperformed those pretrained on ImageNet with the same architecture on the external test set (ResNet50_RadImageNet: AUC = 0.738 [0.714-0.762] vs ResNet50_ImageNet: AUC = 0.669 [0.639–0.699], Z = 4.154, *p* < 0.001; InceptionV3_RadImageNet: AUC = 0.703 [0.674–0.732] vs InceptionV3_ImageNet: AUC = 0.677 [0.647–0.704], Z = 2.543, *p* = 0.026). For models pretrained on RadImageNet, the ResNet50 architecture showed superior performance compared to the InceptionV3 architecture (AUC = 0.738 vs 0.703, Z = 3.954, *p* = 0.010).

A fusion model combining ResNet50_RadImageNet predictions with clinical features (age and tumor location) was developed with the formula:

$${{{\rm{Fusion}}}}\; {{{\rm{score}}}}= \,0.004\times {{{\rm{age}}}}-0.380\times {{{\rm{hand}}}}-0.544\times {{{\rm{foot}}}}\\ + 0.910\times {{{\rm{ResNet}}}}50{\_}{{{\rm{RadImageNet}}}}-0.109$$

**Fig. 3:**
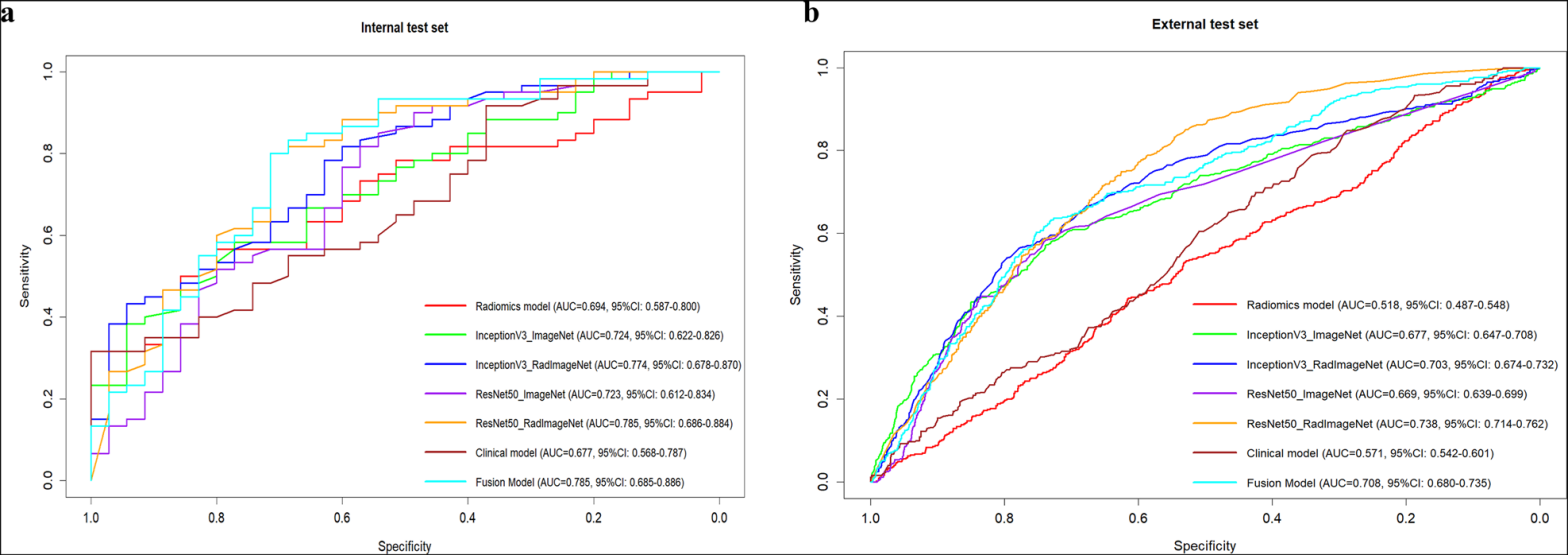
The ROC curves of models in (**a**) internal and (**b**) external test

This fusion model achieved an AUC of 0.785 (95% CI: 0.685–0.886) on the internal test set, identical to the ResNet50_RadImageNet. However, it showed reduced performance on the external test set (AUC = 0.708, 95% CI: 0.680–0.735). The clinical model alone performed poorly across both test sets, particularly on the external cohort (AUC = 0.571, 95% CI: 0.542–0.601). Notably, on the external test set, ResNet50_RadImageNet (AUC = 0.738) significantly outperformed the clinical model (*p* < 0.001). Furthermore, the ResNet50_RadImageNet model also demonstrated superior performance compared to the fusion model (AUC = 0.708, *Z* = 4.045, *p* < 0.001), suggesting that the ResNet50_RadImageNet model, utilizing medical image transfer learning, possesses robust independent predictive capability without relying on clinical data. Figure 3 displays the ROC curves of all models.

To visualize the impact of data imbalance, the model’s performance metrics across different decision thresholds for internal and external datasets were further analyzed. The curves illustrate the trade-offs between sensitivity, specificity, positive predictive value (PPV), and negative predictive value (NPV) as the decision prediction threshold varies from 0 to 1 were also provided in Fig. S4. The prediction threshold corresponding to the maximum Youden Index in training set for ResNet50_RadImageNet model was 0.34, and at this threshold, the model achieved a PPV of 82.8% in the internal test set (prevalence 63.3%); however, in the external test set where the prevalence of malignancy was lower (23.3%), the PPV was 40.8%, which was supported by a maintained specificity of 74.8%.

### Stratified error analysis: subtype, location, and size

To evaluate the clinical robustness of the ResNet50_RadImageNet model, a comprehensive error analysis stratified by tumor subtype, anatomical location, and lesion size was performed.

For tumor subtype (Fig. S5), distinct performance patterns were observed. While the model demonstrated robust detectability for Osteosarcoma (OSA) with a relatively low error rate even in the external set, it struggled with ‘Other’ categories, including “Other Benign” (OBT) and “Other Non-Benign” (ONBT), where error rates exceeded 40-50%. A notable disparity occurred in giant cell tumors (GCT), the error rate surged from 0.0% (internal) to 62.4% (external), with the majority being false negatives.

Regarding anatomical location, performance varied across skeletal regions. The model achieved higher accuracy in the appendicular skeleton (upper/lower limbs), whereas the Axial Skeleton (spine and pelvis) exhibited the highest rate of misclassification (Fig. S6), reflecting the challenge of complex anatomical backgrounds.

For lesion size (Fig. S7), we found that diagnostic sensitivity was significantly correlated with tumor dimensions. In the external dataset, small lesions (Q1, 0–25% percentile) were frequently misclassified, with false negatives (78 cases) outnumbering true positives (54 cases). Conversely, for the largest lesions (Q4, 75–100%), the model demonstrated high sensitivity, with true positives (91 cases) exceeding false negatives (49 cases).

### Robustness analysis of lesion positioning perturbation

**Fig. 4:**
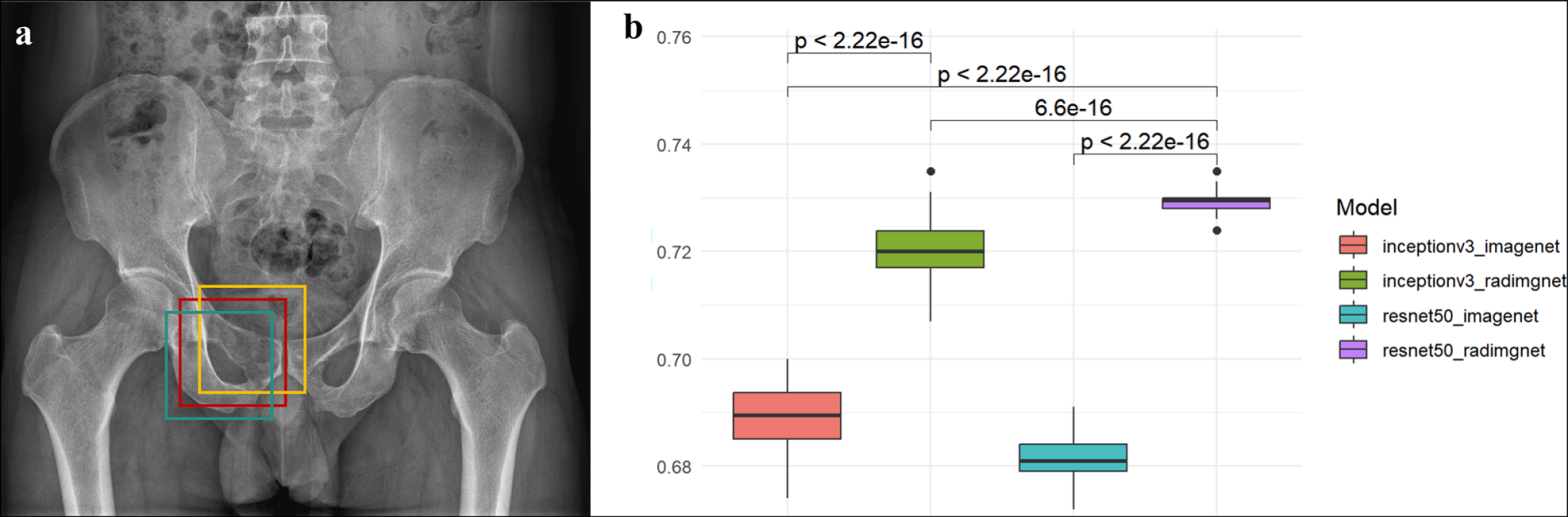
Assessment of the stability of models. **a** Bounding box perturbation experiment. The red bounding box denoting the original position, and the cyan and yellow bounding boxes were randomly displaced by up to ±30% from the original position. **b** The distributions and comparisons of external test AUCs among different models

Model stability was assessed through a bounding box perturbation experiment, where tumor bounding boxes were randomly displaced by up to ±30% from their original positions in 50 iterations. ResNet50_RadImageNet demonstrated superior stability with the highest mean AUC and most concentrated distribution, significantly outperforming other models (*p* < 0.001). Similarly, InceptionV3_RadImageNet significantly outperformed InceptionV3_ImageNet (*p* < 0.001), further confirming that RadImageNet pretraining enhances model stability. Figure 4 presents the distribution of external test AUC values across different models under perturbation conditions.

### Model explainability and auxiliary diagnostic experiment

**Fig. 5:**
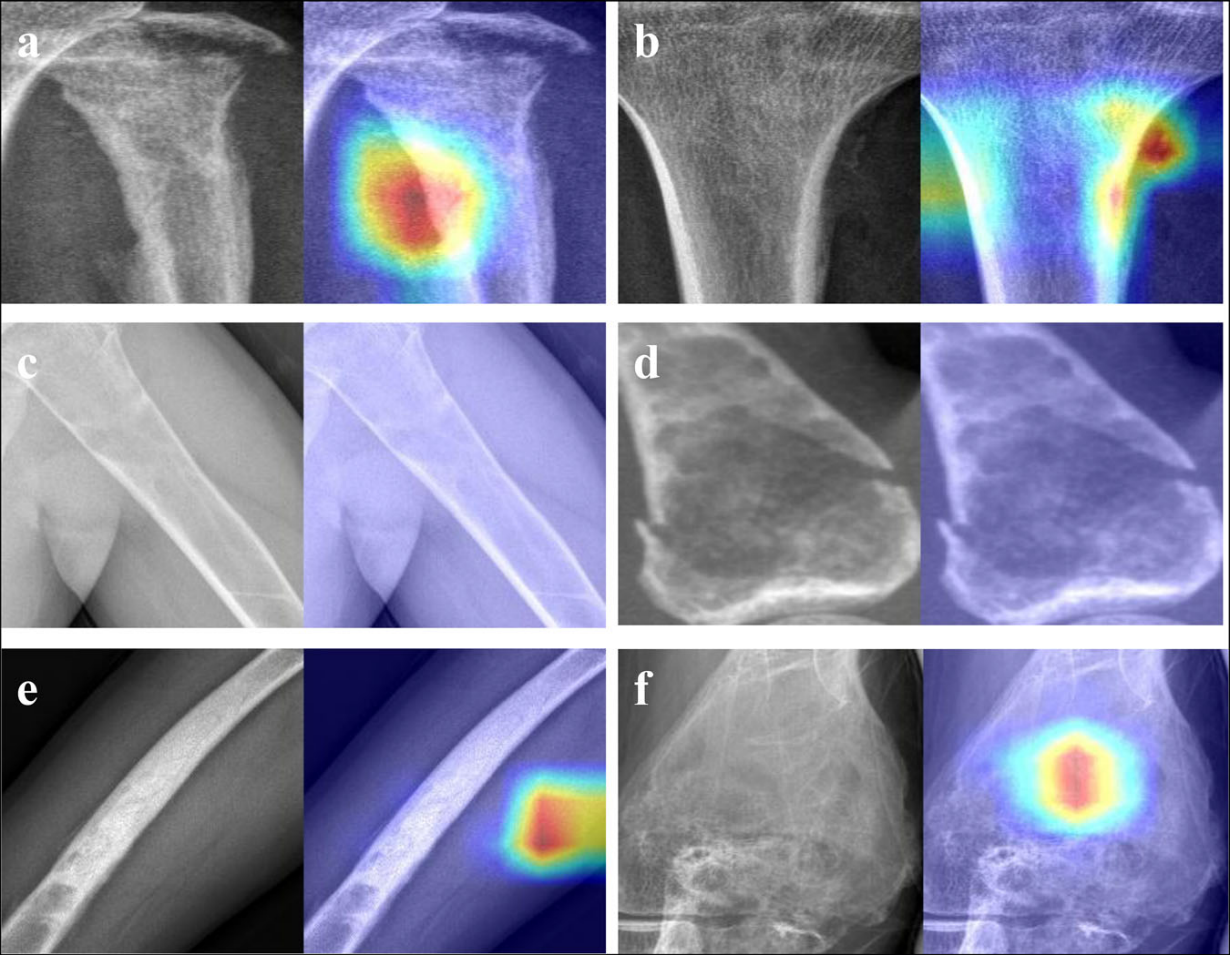
Representative examples of model attention maps. **a** A case of non-benign bone tumor (osteosarcoma) was correctly diagnosed by radiologists and the model. **b** A case of a non-benign bone tumor (osteosarcoma) that was misdiagnosed by radiologists and correctly diagnosed by the model. **c** A case of benign bone tumor (aneurysmal bone cyst) was correctly diagnosed by radiologists and a model. **d** A case of benign bone tumor (endochondroma) was misdiagnosed by radiologists and correctly diagnosed by the model. **e** A case of non-benign bone tumor (osteosarcoma) was misdiagnosed by the model and correctly diagnosed by one radiologist. **f** A case of benign bone tumor (osteofibrous dysplasia) that was misdiagnosed by the model and correctly diagnosed by radiologists

Grad-CAM visualization revealed the attention patterns of ResNet50_RadImageNet. Two radiologists also evaluated the cases independently. Figure 5 presents representative examples of model attention maps and comparative diagnostic assessments with radiologists. The model highlights regions it has predicted as positive, and in correctly classified cases, the model appropriately focused on diagnostically relevant regions. For example, in the case of osteosarcoma, the model correctly identified non-benign characteristics that aligned with radiologists’ assessments, as the model’s attention regions concentrated on the transition zone between the tumor and normal tissue, where blurred boundaries caused by aggressive cortical destruction and permeative growth into surrounding soft tissues represent common radiological signs of malignancy. Interestingly, in cases like enchondroma, the model correctly classified the tumor while radiologists made incorrect assessments. Meanwhile, in cases where the model made incorrect classifications, the highlighted regions failed to focus on diagnostically relevant areas.

**Fig. 6:**
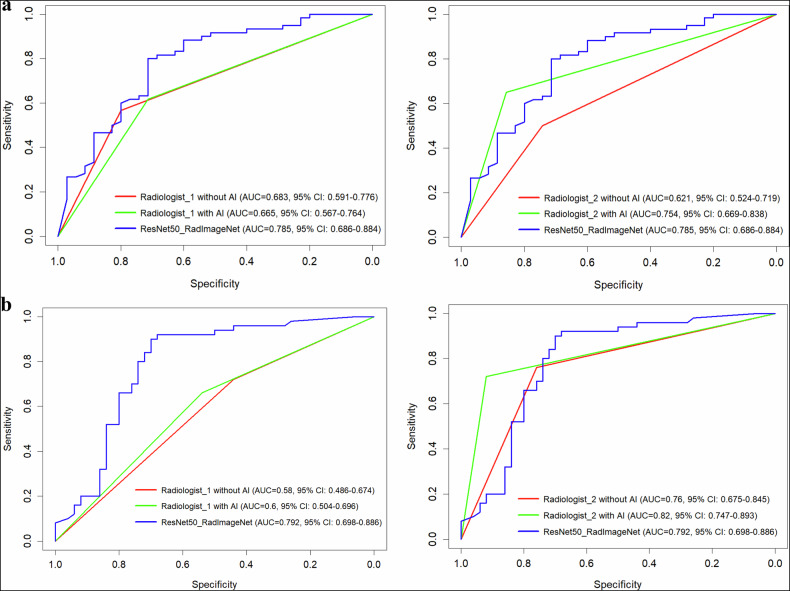
The ROC curves of radiologists with and without model assistance. (**a**) Internal test. (**b**) External test

Two radiologists independently evaluated the cases both with and without model assistance (Fig. 6). At baseline (without AI), Radiologist_1 and Radiologist_2 demonstrated comparable performance on the internal test set (AUC: 0.683 [0.591–0,776] vs 0.621 [0.524–0.719], *Z* = 1.362, *p* = 0.173). However, on the external test set, a significant performance gap was observed: the more experienced Radiologist_2 outperformed Radiologist_1 (AUC: 0.760 [0.675–0.845] vs 0.580 [0.486–0.674], *Z* = 4.937, *p* < 0.001), reflecting the superior generalizability often associated with greater clinical experience.

Regarding the impact of AI assistance, the results varied by reader experience. For Radiologist 1, AI assistance did not significantly improve diagnostic performance on the internal test set (AUC = 0.665 [95% CI: 0.567–0.764] vs 0.683 [0.591–0.776]; difference = −0.018 [95% CI: −0.073 to 0.037], *Z* = −0.640, *p* = 0.522) or the external test set (AUC = 0.600 vs 0.580; difference = 0.020 [−0.033 to 0.074], *Z* = 0.732, *p* = 0.464). Notably, the upper bound of the CI for the internal set (0.037) excluded a clinically substantial benefit (ΔAUC > 0.05). In contrast, Radiologist 2 derived substantial benefit from the model, achieving a performance boost on the internal test set (AUC = 0.754 [0.669–0.838] vs 0.621 [0.524-0.719]; difference = 0.133 [0.049 to 0.218], *Z* = 3.076, *p* = 0.002) and showing a trend toward improvement on the external test set (AUC = 0.820 [0.747–0.893] vs 0.760 [0.675–0.845]; difference = 0.060 [−0.005 to 0.125], *Z* = 1.821, *p* = 0.068).

## Discussion

This multi-center study demonstrated the superior performance of DL models over radiomics approaches for bone tumor classification on radiographs. Our results specifically highlight the significant advantages of domain-specific medical image pretraining in improving the classification performance and stability, with important implications for potential support in a supervised diagnostic workflow.

For the radiomics model, a predominance of wavelet-transformed features (8 out of 11 selected features) was observed. This finding is consistent with previous radiomics studies on bone tumors, where wavelet features have been shown to constitute the majority of selected features and play a critical role in capturing multi-scale texture information that is biologically relevant for characterizing malignant tissue alterations [25, 26]. However, the radiomics model achieved only moderate performance on internal validation (AUC = 0.688) and poor performance on external validation (AUC = 0.524). This substantial performance drop indicates limited generalizability, while only a few studies applied external data sets for validation[27]. The poor generalizability of radiomics may be due to the fact that the handcrafted features are highly sensitive to variations in image acquisition parameters and tumor delineations [28], limiting their potential assistive value in multi-center settings. This hypothesis is supported by our quantitative analysis, which revealed a substantial domain shift, particularly in image contrast. Such a contrast shift, likely arising from inter-site acquisition heterogeneity and vendor-specific post-processing, creates a challenging testing environment. However, all DL models demonstrated substantially better generalizability, maintaining reasonable performance on external validation. This suggests that learned feature representations may capture more robust and transferable imaging characteristics compared to predefined radiomics features.

The RadImageNet pretrained model outperformed the ImageNet pretrained model, and this pattern held true across different architectures. This performance advantage aligns with previous research by Mei et al [12], who first introduced RadImageNet and demonstrated its superiority over ImageNet for various radiological applications. The 10.3% improvement we observed in external validation AUC when using RadImageNet vs ImageNet pretraining (0.738 vs 0.669) is particularly noteworthy, and it was similar with the findings of other studies, for example, Zhang et al [29] reported a 8.4% AUC improvement in external validation for vertebral fracture classification on radiographs, suggesting that the generalization ability of radiographs classification model may particularly benefit from medical image pretraining. The superior performance likely stems from RadImageNet’s closer alignment with the medical image domain, and the dataset comprises radiological images sharing similar grayscale appearances, contrast patterns, and anatomical structures with bone tumor radiographs. This domain similarity facilitates more effective knowledge transfer, as the model has already learned relevant feature representations during pretraining. Furthermore, our results demonstrated that the ResNet50 architecture outperformed InceptionV3 with RadImageNet pretraining, and it was consistent with a previous study [12]. It may be attributed to its residual connections that facilitate training deeper networks while mitigating the vanishing gradient problem [18]. This architecture appears particularly advantageous for bone tumor classification, where subtle textural and edge features at multiple scales are diagnostically important [7]. Beyond just performance metrics, our bounding box perturbation experiment revealed that RadImageNet-pretrained models demonstrated significantly better stability compared to ImageNet-pretrained counterparts. This enhanced robustness to variations in tumor localization is critical for clinical deployment, where precise tumor segmentation may not always be available.

The fusion model combining ResNet50_RadImageNet predictions with clinical features (age and tumor location) achieved the highest AUC (0.788) on internal validation but showed reduced performance (AUC = 0.699) on external validation, underperforming the pure imaging model (AUC = 0.738). Contrary to the previous single-center validation study [10], this finding suggests that the relationship between clinical features and tumor classification may vary across different patient populations, potentially introducing bias rather than enhancing generalizability. Similar findings have been reported in several multicenter studies, where fusion models failed to yield superior performance during external testing [30].

The stratified analysis reveals that misclassifications are not random but driven by specific radiographic and anatomical challenges. The high false-negative rate for GCT in the external set suggests a specific pitfall where the model interprets their appearing features as a non-aggressive lesion. Conversely, osteosarcoma was robustly identified, likely because its high-grade malignant features are distinct and domain-invariant. The reduced performance in the axial skeleton highlights the difficulty of isolating lesions from complex overlapping structures (e.g., bowel gas, ribs) compared to the cleaner background of the appendicular skeleton. The frequent misclassification of small lesions indicates that these tumors may lack the conspicuous destructive features required for confident AI identification. These findings suggest that radiologists should exercise increased vigilance when interpreting small, lytic lesions in the axial skeleton, as these scenarios represent the model’s primary blind spots.

Furthermore, it is important to address the class imbalance and distribution shift between our study datasets and real-world clinical practice. Our internal dataset was enriched with non-benign tumors (63.3%), reflecting a tertiary referral center, while the external test set had a lower prevalence (23.3%). This prevalence difference resulted in a lower PPV (40.8%) in the external set compared to the internal set (82.8%). However, the model maintained a specificity of 74.8%. This characteristic is particularly relevant for the model’s application in general practice or primary care settings, where benign bone lesions are frequent incidental findings. High specificity is crucial to prevent “referral overload” at specialized centers caused by false positives. Therefore, despite the lower PPV driven by low prevalence, the model may serve as a valuable triage decision support tool, helping effectively filter benign cases while flagging suspicious lesions for specialist review.

The visualization of model attention patterns showed that ResNet50_RadImageNet appropriately focused on diagnostically relevant regions in correctly classified cases, further enhancing the interpretability and rationality of the model, which was also useful in aiding clinicians by rapidly drawing attention to relevant regions. The cases where the model correctly classified tumors that radiologists misdiagnosed highlight the potential complementary role of AI in clinical practice.

The auxiliary diagnostic experiment provided further insights into the interaction between radiologist experience and AI assistance. While both readers were attending musculoskeletal radiologists, the more senior reader demonstrated significantly superior generalizability on the external test set compared to the less experienced reader. This aligns with the expectation that extensive clinical experience fosters better robustness against domain shifts in unseen data. However, on the internal test set, the more senior reader’s baseline performance was subject to variability, statistically comparable to, yet numerically lower than that of a less experienced reader. Crucially, AI assistance proved most beneficial here, significantly boosting the senior reader’s performance. This indicates that while expert radiologists possess strong generalization skills, they are still susceptible to inter-observer variability or fatigue-related fluctuations. In this context, the model functioned effectively as a ‘second-reader safety net’, calibrating the expert’s judgment and mitigating performance dips. Conversely, the lack of significant improvement for less experienced readers suggests that less experienced readers might face challenges in effectively integrating AI predictions, possibly due to confirmation bias or uncertainty in resolving disagreements with the model. This differential impact echoes previous findings that AI assistance improved diagnostic accuracy for some radiologists but not others, depending on their experience level and receptiveness to AI support [31–33].

Building on these insights, the practical position of the model within a clinical setting is best defined as a semi-automated, supervised workflow, rather than a fully autonomous screening pipeline. In this interactive setup, the radiologist first identifies and delineates the lesion bounding box, and the model instantly provides a prediction to assist in characterization. For specialized centers (high prevalence of malignancy), the model may be used for experts to mitigate fatigue-related errors. For primary care or general practice (high prevalence of benign lesions), the model may serve as a triage tool. Furthermore, its robust specificity (74.8% even in external sets) supports the confident dismissal of benign lesions, potentially reducing unnecessary biopsies.

The main limitations of our study are as follows: first, the binary classification approach (benign vs non-benign) rather than specific histological diagnoses. We acknowledge that this simplifies the pathological complexity of bone tumors, particularly regarding intermediate entities. Additionally, the exclusive focus on radiographs, despite clinical practice typically incorporating multiple imaging modalities. Future research should explore multi-class classification for specific tumor subtypes and the integration of multiple imaging modalities. Third, we acknowledge that while the model achieved high specificity, the sensitivity in the external test set decreased to 57.1% compared to the internal set. This drop in sensitivity is a known challenge in DL applications, often attributed to domain shift (differences in imaging protocols and scanners) and spectrum bias (heterogeneity in tumor subtypes between hospitals). Consequently, in its current form, the model may be utilized as a triage support tool in general clinical practice. Future studies involving multi-center training data and techniques such as domain adaptation are necessary to improve the model’s sensitivity and generalizability across diverse clinical settings. Furthermore, variability in manual segmentation protocols across different centers, may introduce noise and affect radiomics feature stability. Future work could benefit from employing automated or semi-automated segmentation tools to enhance reproducibility.

In conclusion, our study demonstrates that DL models pretrained on RadImageNet outperform both radiomics approaches and models pretrained on ImageNet for bone tumor classification on radiographs. The RadImageNet pretrained ResNet50 model shows superior performance and stability compared to others, and AI assistance can improve radiologist performance in some cases. These findings support the value of domain-specific pretraining and highlight the preliminary feasibility of the developed model as a diagnostic support tool for bone tumor evaluation.

## Supplementary information

ELECTRONIC SUPPLEMENTARY MATERIAL

## Data Availability

Some study cohorts from BTXRD have been previously reported in Scientific Data (2025) 12:88 (10.1038/s41597-024-04311-y). The other datasets underlying this article will be shared on reasonable request to the corresponding author.

## Abbreviations

AUC: Area under the receiver operating characteristic curve
BTXRD: Bone tumor X-ray radiograph dataset
DL: Deep learning
Grad-CAM: Gradient-weighted class activation mapping

## References

[1] Hosseini H, Heydari S, Hushmandi K, Daneshi S, Raesi R (2025) Bone tumors: a systematic review of prevalence, risk determinants, and survival patterns. BMC Cancer 25:321 39984867 10.1186/s12885-025-13720-0 PMC11846205

[2] De Salvo S, Pavone V, Coco S, Dell’Agli E, Blatti C, Testa G (2022) Benign bone tumors: an overview of what we know today. J Clin Med 11:699 10.3390/jcm11030699 PMC8836463 35160146

[3] Marina NM, Smeland S, Bielack SS et al (2016) Comparison of MAPIE versus MAP in patients with a poor response to preoperative chemotherapy for newly diagnosed high-grade osteosarcoma (EURAMOS-1): an open-label, international, randomised controlled trial. Lancet Oncol 17:1396–1408 27569442 10.1016/S1470-2045(16)30214-5 PMC5052459

[4] Tomasian A, Hillen TJ, Jennings JW (2020) Bone biopsies: what radiologists need to know. AJR Am J Roentgenol 215:523–533 32755186 10.2214/AJR.20.22809

[5] Casali PG, Bielack S, Abecassis N et al (2018) Bone sarcomas: ESMO-PaedCan-EURACAN clinical practice guidelines for diagnosis, treatment and follow-up. Ann Oncol 29:iv79–iv95 30285218 10.1093/annonc/mdy310

[6] Franchi A (2012) Epidemiology and classification of bone tumors. Clin Cases Miner Bone Metab 9:92–95 23087718 PMC3476517

[7] Costelloe CM, Madewell JE (2013) Radiography in the initial diagnosis of primary bone tumors. AJR Am J Roentgenol 200:3–7 23255735 10.2214/AJR.12.8488

[8] Xie Z, Zhao H, Song L et al (2024) A radiograph-based deep learning model improves radiologists’ performance for classification of histological types of primary bone tumors: a multicenter study. Eur J Radiol 176:111496 38733705 10.1016/j.ejrad.2024.111496

[9] von Schacky CE, Wilhelm NJ, Schafer VS et al (2021) Multitask deep learning for segmentation and classification of primary bone tumors on radiographs. Radiology 301:398–406 34491126 10.1148/radiol.2021204531

[10] Liu R, Pan D, Xu Y et al (2022) A deep learning-machine learning fusion approach for the classification of benign, malignant, and intermediate bone tumors. Eur Radiol 32:1371–1383 34432121 10.1007/s00330-021-08195-z

[11] Lacroix M, Aouad T, Feydy J et al (2023) Artificial intelligence in musculoskeletal oncology imaging: a critical review of current applications. Diagn Interv Imaging 104:18–23 36270953 10.1016/j.diii.2022.10.004

[12] Mei X, Liu Z, Robson PM et al (2022) RadImageNet: an open radiologic deep learning research dataset for effective transfer learning. Radiol Artif Intell 4:e210315 36204533 10.1148/ryai.210315 PMC9530758

[13] Gitto S, Cuocolo R, Albano D et al (2021) CT and MRI radiomics of bone and soft-tissue sarcomas: a systematic review of reproducibility and validation strategies. Insights Imaging 12:68 34076740 10.1186/s13244-021-01008-3 PMC8172744

[14] Gitto S, Cuocolo R, Huisman M et al (2024) CT and MRI radiomics of bone and soft-tissue sarcomas: an updated systematic review of reproducibility and validation strategies. Insights Imaging 15:54 38411750 10.1186/s13244-024-01614-x PMC10899555

[15] Yao S, Huang Y, Wang X et al (2025) A radiograph dataset for the classification, localization, and segmentation of primary bone tumors. Sci Data 12:88 39820508 10.1038/s41597-024-04311-y PMC11739492

[16] Sbaraglia M, Bellan E, Dei Tos AP (2021) The 2020 WHO classification of soft tissue tumours: news and perspectives. Pathologica 113:70–84 33179614 10.32074/1591-951X-213 PMC8167394

[17] Yushkevich PA, Piven J, Hazlett HC et al (2006) User-guided 3D active contour segmentation of anatomical structures: significantly improved efficiency and reliability. Neuroimage 31:1116–1128 16545965 10.1016/j.neuroimage.2006.01.015

[18] He K, Zhang X, Ren S, Sun J (2016) Deep residual learning for image recognition. In: Proceedings of the IEEE conference on computer vision and pattern recognition. IEEE, Las Vegas, pp 770–778

[19] Szegedy C, Vanhoucke V, Ioffe S, Shlens J, Wojna Z (2016) Rethinking the inception architecture for computer vision. In: Proceedings of the IEEE conference on computer vision and pattern recognition. IEEE, pp 2818–2826

[20] Deng J, Dong W, Socher R, Li L-J, Li K, Fei-Fei L (2009) Imagenet: a large-scale hierarchical image database. 2009 IEEE conference on computer vision and pattern recognition. CVPR, Florida, pp 248–255

[21] Lin TY, Goyal P, Girshick R, He K, Dollar P (2020) Focal loss for dense object detection. IEEE Trans Pattern Anal Mach Intell 42:318–327 30040631 10.1109/TPAMI.2018.2858826

[22] van Griethuysen JJM, Fedorov A, Parmar C et al (2017) Computational radiomics system to decode the radiographic phenotype. Cancer Res 77:e104–e107 29092951 10.1158/0008-5472.CAN-17-0339 PMC5672828

[23] Zou H, Hastie T (2005) Regularization and variable selection via the elastic net. J Royal Stat Soc Stat Methodol 67:301–320

[24] Selvaraju RR, Cogswell M, Das A, Vedantam R, Parikh D, Batra D (2017) Grad-CAM: visual explanations from deep networks via gradient-based localization. In: Proceedings of the IEEE international conference on computer vision. IEEE, Venice, pp 618–626

[25] Gao Z, Dai Z, Ouyang Z et al (2024) Radiomics analysis in differentiating osteosarcoma and chondrosarcoma based on T2-weighted imaging and contrast-enhanced T1-weighted imaging. Sci Rep 14:26594 39496777 10.1038/s41598-024-78245-1 PMC11535035

[26] von Schacky CE, Wilhelm NJ, Schafer VS et al (2022) Development and evaluation of machine learning models based on X-ray radiomics for the classification and differentiation of malignant and benign bone tumors. Eur Radiol 32:6247–6257 35396665 10.1007/s00330-022-08764-w PMC9381439

[27] Fritz B, Yi PH, Kijowski R, Fritz J (2023) Radiomics and deep learning for disease detection in musculoskeletal radiology: an overview of novel MRI- and CT-based approaches. Invest Radiol 58:3–13 36070548 10.1097/RLI.0000000000000907

[28] Park JE, Park SY, Kim HJ, Kim HS (2019) Reproducibility and generalizability in radiomics modeling: possible strategies in radiologic and statistical perspectives. Korean J Radiol 20:1124–1137 31270976 10.3348/kjr.2018.0070 PMC6609433

[29] Zhang J, Xia L, Tang J et al (2024) Constructing a deep learning radiomics model based on X-ray images and clinical data for predicting and distinguishing acute and chronic osteoporotic vertebral fractures: a multicenter study. Acad Radiol 31:2011–2026 38016821 10.1016/j.acra.2023.10.061

[30] Demircioglu A (2023) Are deep models in radiomics performing better than generic models? A systematic review. Eur Radiol Exp 7:11 36918479 10.1186/s41747-023-00325-0 PMC10014394

[31] Xia W, Li D, He W et al (2024) Multicenter evaluation of a weakly supervised deep learning model for lymph node diagnosis in rectal cancer at MRI. Radiol Artif Intell 6:e230152 38353633 10.1148/ryai.230152 PMC10982819

[32] Yu F, Moehring A, Banerjee O, Salz T, Agarwal N, Rajpurkar P (2024) Heterogeneity and predictors of the effects of AI assistance on radiologists. Nat Med 30:837–849 38504016 10.1038/s41591-024-02850-w PMC10957478

[33] Dratsch T, Chen X, Rezazade Mehrizi M et al (2023) Automation bias in mammography: the impact of artificial intelligence BI-RADS suggestions on reader performance. Radiology 307:e222176 37129490 10.1148/radiol.222176

