# Supplementary material for: Medical image pretraining-based transfer learning for generalizable and robust diagnosis of bone tumors on radiographs: a multi-center study
Source: Insights Imaging. 2026 Apr 7;17:94. doi: 10.1186/s13244-026-02271-y (PMC13057043; doi:10.1186/s13244-026-02271-y)
Supplement: Supplementary file 1 — ELECTRONIC SUPPLEMENTARY MATERIAL [file 13244_2026_2271_MOESM1_ESM.pdf]

Medical Image Pretraining based Transfer Learning for Generalizable and Robust Diagnosis of Bone Tumors on Radiographs: A Multi-Center Study

ELECTRONIC SUPPLEMENTARY MATERIAL

Table S1. Image histogram distributions of internal and external datasets across four metrics including mean intensity, contrast (pixel standard deviation), entropy, and aspect ratio.

Image metrics	Internal dataset	External dataset	Cohen's d	P
Mean Intensity	0.264 ± 0.121	0.268 ± 0.097	0.04	0.468
Contrast	0.151 ± 0.047	0.215 ± 0.052	1.30	<0.001
Entropy	6.325 ± 0.703	6.246 ± 0.842	-0.10	0.032
Aspect Ratio	0.750 ± 0.241	0.801 ± 0.273	0.20	<0.001

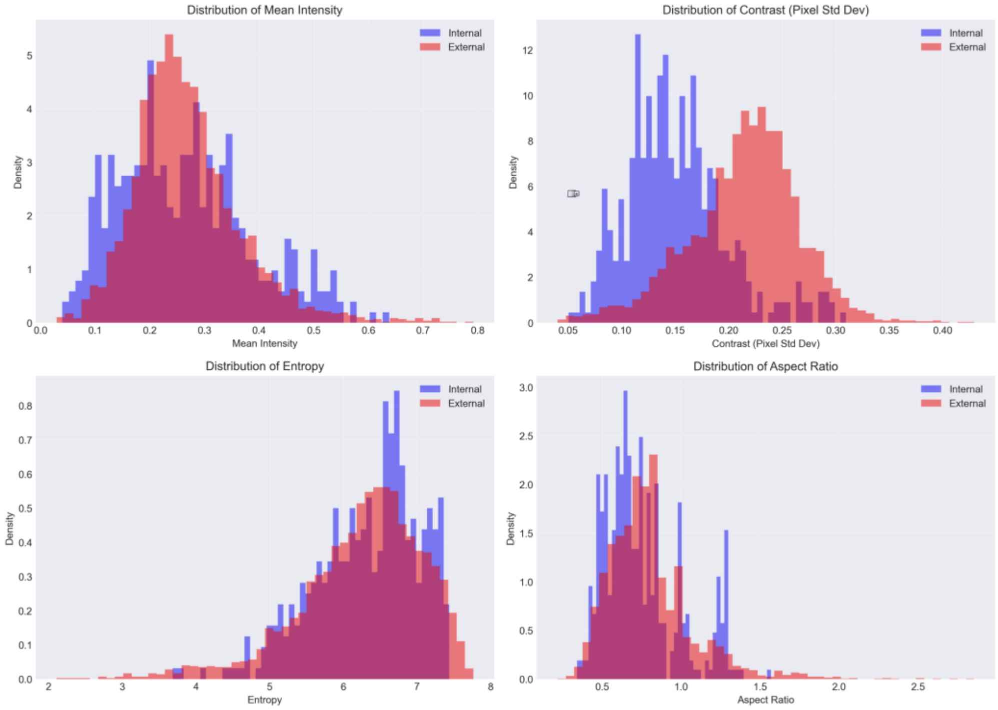

Figure S1. The image histogram distributions of internal and external datasets across mean intensity, contrast (pixel standard deviation), entropy, and aspect ratio.

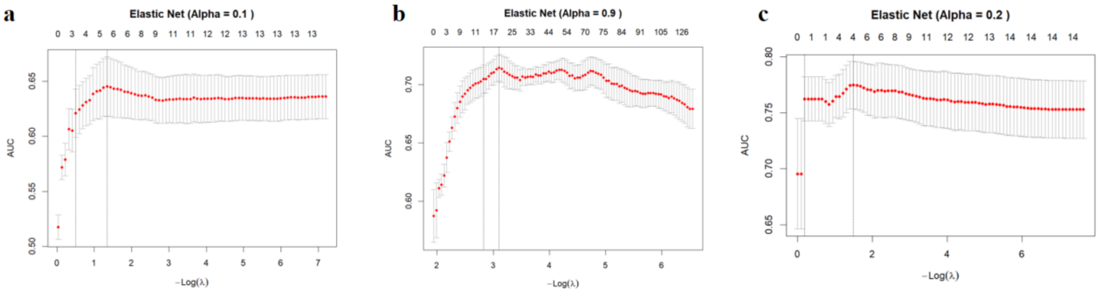

Figure S2. Cross-validation performance curves for ElasticNet regularization parameter optimization. AUC values across different alpha values (0.1-0.9) for (a) clinical, (b) radiomics, and (c) fusion modeling using 10-fold cross-validation. The optimal alpha values were selected based on maximum mean AUC performance.

Table S2. Selected radiomic features and their corresponding coefficients in the optimal ElasticNet model. Features were automatically selected through ElasticNet regularization during 10-fold cross-validation.

Feature name	Coefficient
original_firstorder_Energy	1.560*10-06
original_gldm_SmallDependenceEmphasis	-1.364
original_gldm_SmallDependenceHighGrayLevelEmphasis	-5.950
wavelet.HL_glcm_JointAverage	36.684
wavelet.HL_glcm_ClusterShade	-3.453
wavelet.HL_gldm_LowGrayLevelEmphasis	-1.741
wavelet.HL_gldm_SmallDependenceHighGrayLevelEmphasis	-1.375
wavelet.HL_glszm_SmallAreaLowGrayLevelEmphasis	0.888
wavelet.LL_firstorder_Energy	3.462
wavelet.LL_glrlm_HighGrayLevelRunEmphasis	-0.694
wavelet.LL_glrlm_LowGrayLevelRunEmphasis	2.324
Intercept	-53.413

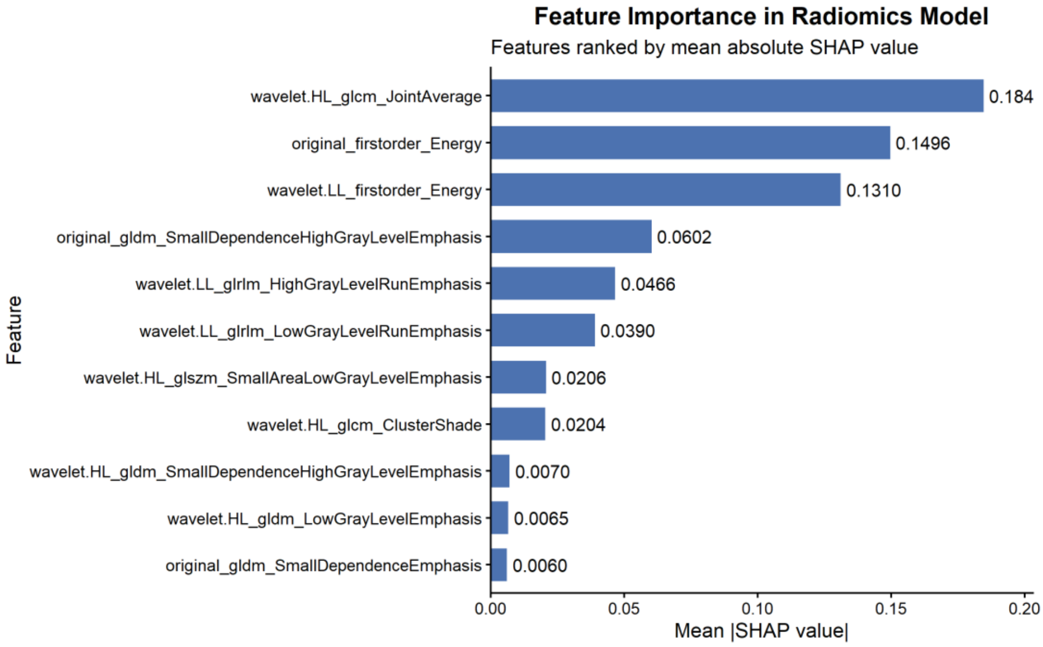

Figure S3. Feature importance analysis for the radiomics ElasticNet model based on SHAP (SHapley Additive exPlanations) values. Mean absolute SHAP values for features selected by the ElasticNet model, ranked by their contribution to model predictions. SHAP values quantify the marginal contribution of each feature to the model output, with higher values indicating greater importance.

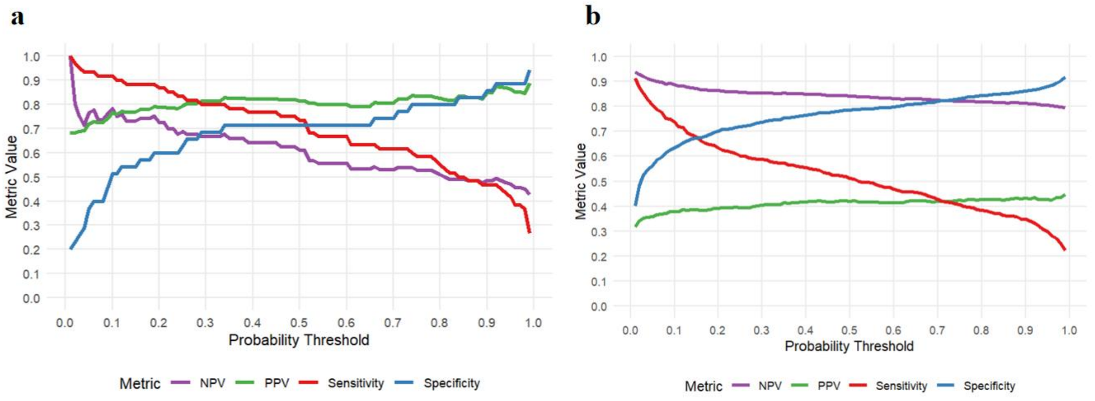

Figure S4. Threshold-dependent performance analysis of the deep learning model ResNet50_RadImageNet across datasets. The curves illustrate the trade-offs between Sensitivity (red), Specificity (blue), Positive Predictive Value (PPV, green), and Negative Predictive Value (NPV, purple) as the decision probability threshold varies from 0 to 1. (a) Internal Test Set (High Prevalence, 63.3%). (b) External Test Set (Low Prevalence, 23.3%).

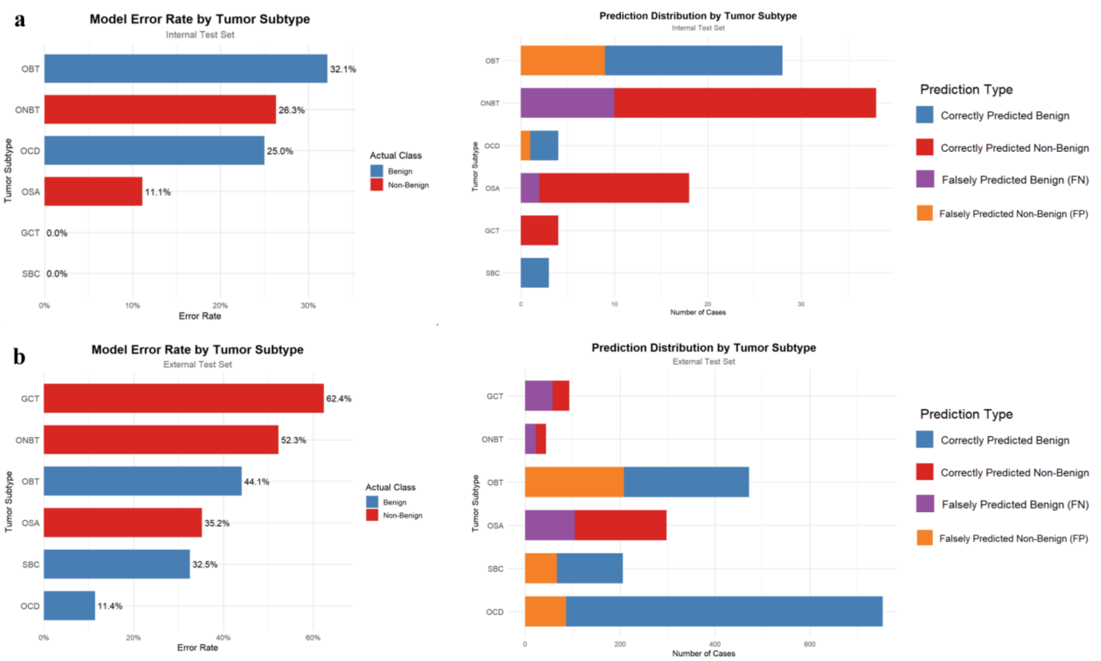

Figure S5. Comprehensive Error Analysis by Tumor Subtype. (a) Performance on the Internal Test Set. (b) Performance on the External Test Set. The left panels display the model error rate (%) for each tumor subtype. Blue bars indicate Benign subtypes, and Red bars indicate Non-Benign subtypes. The right panels illustrate the absolute distribution of prediction outcomes. Note. OCD = Osteochondroma, SBC = Simple Bone Cyst, OBT = Other Benign Tumor, GCT = Giant Cell Tumor, OSA = Osteosarcoma, ONBT = Other Non-Benign Tumor.

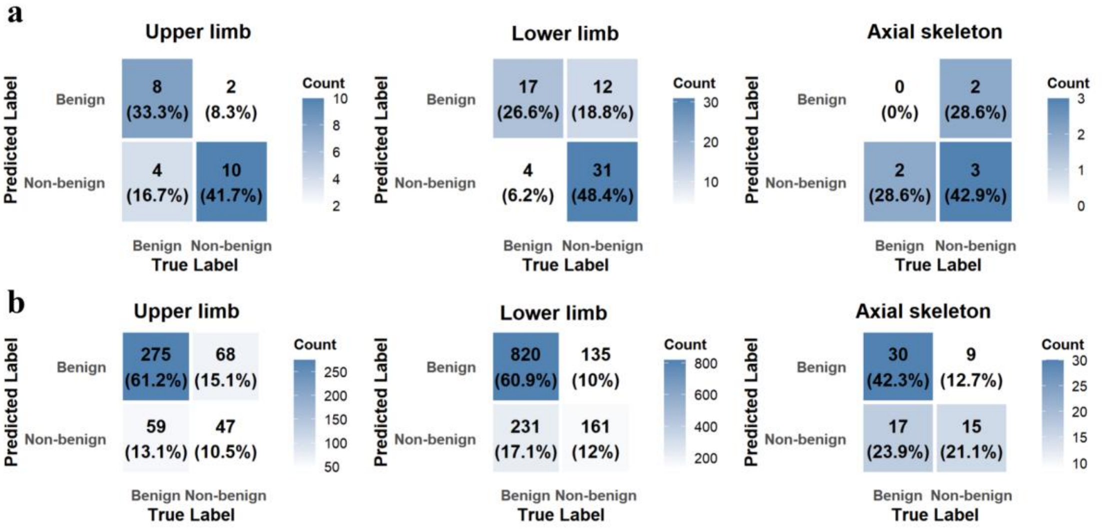

Figure S6. Stratified Confusion Matrices by Anatomical Region. (a) Internal Test Set performance across Upper Limb, Lower Limb, and Axial Skeleton. (b) External Test Set performance across the same regions. Each matrix displays the count and percentage of predictions relative to the total number of cases in that specific subgroup.

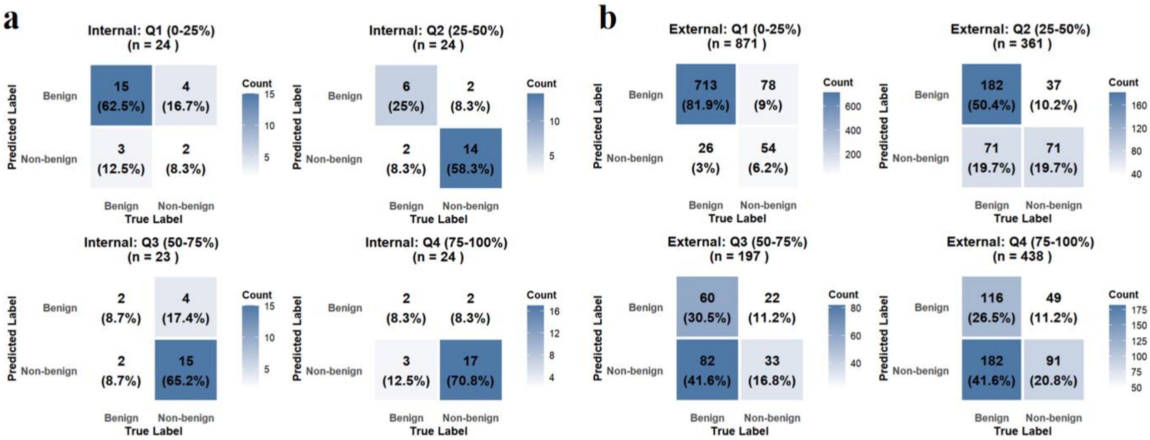

Figure S7. Stratified Confusion Matrices by Lesion Size Quartiles. Performance of the model on the (a) Internal Test Set and (b) External Test Set, stratified by lesion size (bounding box pixel area) into four quartiles: Q1 (Smallest, 0–25%) to Q4 (Largest, 75–100%) derived from the internal dataset's lesion size distribution.
